# Dietary Mechanism behind the Costs Associated with Resistance to *Bacillus thuringiensis* in the Cabbage Looper, *Trichoplusia ni*

**DOI:** 10.1371/journal.pone.0105864

**Published:** 2014-08-29

**Authors:** Ikkei Shikano, Jenny S. Cory

**Affiliations:** Department of Biological Sciences, Simon Fraser University, Burnaby, British Columbia, Canada; Ghent University, Belgium

## Abstract

Beneficial alleles that spread rapidly as an adaptation to a new environment are often associated with costs that reduce the fitness of the population in the original environment. Several species of insect pests have evolved resistance to *Bacillus thuringiensis* (*Bt*) toxins in the field, jeopardizing its future use. This has most commonly occurred through the alteration of insect midgut binding sites specific for *Bt* toxins. While fitness costs related to *Bt* resistance alleles have often been recorded, the mechanisms behind them have remained obscure. We asked whether evolved resistance to *Bt* alters dietary nutrient intake, and if reduced efficiency of converting ingested nutrients to body growth are associated with fitness costs and variation in susceptibility to *Bt*. We fed the cabbage looper *Trichoplusia ni* artificial diets differing in levels of dietary imbalance in two major macronutrients, protein and digestible carbohydrate. By comparing a *Bt*-resistant *T. ni* strain with a susceptible strain we found that the mechanism behind reduced pupal weights and growth rates associated with *Bt*-resistance in *T. ni* was reduced consumption rather than impaired conversion of ingested nutrients to growth. In fact, *Bt*-resistant *T. ni* showed more efficient conversion of nutrients than the susceptible strain under certain dietary conditions. Although increasing levels of dietary protein prior to *Bt* challenge had a positive effect on larval survival, the LC_50_ of the resistant strain decreased when fed high levels of excess protein, whereas the LC_50_ of the susceptible strain continued to rise. Our study demonstrates that examining the nutritional basis of fitness costs may help elucidate the mechanisms underpinning them.

## Introduction

Repeated use of chemical insecticides, as well as several microbial insecticides, has resulted in the evolution of resistance in numerous insect species [1]–[5]. However, the mutations that confer resistance often reduce fitness in the absence of the insecticide [6]–[10]. Hence, the evolution and stability of resistance to microbial and chemical insecticides is believed to be strongly influenced by fitness costs [11], .

The toxin-producing bacterium *Bacillus thuringiensis* (*Bt*), is the most commercially successful microbial insecticide, and crop plants genetically modified to express *Bt* toxins have been planted in 66 million hectares worldwide [13]. Repeated exposure to *Bt* has placed strong selection pressure on its target herbivores, resulting in some instances in the evolution of resistance [3], [5], [14]. Resistance in most species decreases rapidly in the absence of *Bt* exposure, suggesting a trade-off in which alleles for *Bt* resistance increase fitness in the presence of *Bt* but inflict fitness costs in its absence [15]. Fitness costs associated with resistance to *Bt* sprays or *Bt* toxins have been found in representatives from one family of Coleoptera (Chrysomelidae [16]) and Diptera (Culicidae [17]), and four families of Lepidoptera (Noctuidae [18], [19], Plutellidae [20], [21], Pyralidae [22], and Gelechiidae [23]).

Mortality from *Bt* occurs when *Bt* Cry toxins are ingested, either contained within *Bt* spores or expressed in transgenic crops, and interact with specific binding sites at the midgut brush border membrane, forming pores that result in cell lysis and septicemia, causing death of the insect [24], [25]. The evolution of *Bt* resistance is typically defined as a genetically based reduction in the susceptibility of a population to *Bt* Cry toxins [5], and most commonly involves reduced binding of the toxins to midgut targets through the alteration or loss of midgut toxin-binding proteins [5]. Several other toxin-based resistance mechanisms have been found including sequestration of the toxin by lipophorin [26], esterases [27] or alkaline phosphatase [28]. However, resistance has also been shown to occur through elevated immune responses to formulations containing *Bt* spores [29], [30], which invade the haemocoel after the toxins breach the intestinal epithelium [31], [32]. The *Bt*-resistant strain of *Trichoplusia ni* (Hübner), used in the present study was originally collected from a vegetable greenhouse and found to be highly resistant to DiPel [19], a formulation of spores and toxins of *B. thuringiensis* subsp. *kurstaki* containing Cry1Aa, Cry1Ab, Cry1Ac, and Cry2. It has since been routinely selected for resistance to DiPel, but the mechanism of resistance in this strain is unknown.

Fitness costs associated with *Bt*-resistance are strongly influenced by ecological variation, such as the plants the insects feed on [21], [33]–[38], and are magnified by defensive phytochemicals that reduce feeding performance through direct toxic effects or reduced availability of nutrients [15]. In our *Bt*-resistant strain of *T. ni*, the degree of resistance-associated fitness costs (lower pupal weight and slower development rate) increased with declining host plant suitability [33], [39]. We hypothesized that these costs could be caused by an impaired ability to convert ingested nutrients into body growth. We have also recently shown that the *Bt*-resistant strain selects a higher ratio of protein to carbohydrate than the susceptible strain when they are allowed to compose their own protein to carbohydrate diet [40]. The intake of more protein could indicate compensatory feeding to overcome impaired conversion of ingested protein into bodily nitrogen or increased protein requirements to maintain an elevated immune response [41],[42].

Nutritional studies have shown that insects regulate their nutrient intake to optimize performance [43], [44]. In order to examine the mechanisms behind the fitness costs associated with resistance to *Bt*, we used a well-established Geometric Framework from nutritional ecology [45]–[47] to compare *Bt*-resistant and *Bt*-susceptible lines of cabbage loopers, *Trichoplusia ni*. This approach quantifies how insects regulate the intake of two or more food components at the same time [47], [48]. We restricted *Bt*-resistant and *Bt*-susceptible lines of *T. ni* to artificial diets differing in levels of dietary imbalance in two major macronutrients, protein and digestible carbohydrate to answer the following questions: (i) Do changes in nutrient availability affect the presence or level of costs associated with *Bt* resistance? (ii) Does the evolution of resistance to *Bt* affect nutrient intake? (iii) Is there evidence for impaired nutrient use in resistant insects? (iv) Do the observed fitness costs result from reduced nutrient intake or conversion efficiency? Lastly, (v) How does nutrient availability affect *Bt*-resistance? As reductions in pupal mass and growth rate, and delayed time to pupation have been shown to be fitness costs in *Bt*-resistant *T. ni* and other *Bt*-resistant lepidopterans [15], we chose these as our metrics to assess how nutrient availability affects fitness costs.

## Materials and Methods

### Study Animals

The *Bt*-resistant *T. ni* colony has been maintained at 25°C and 16∶8 (L∶D) photoperiod on a standard wheat-germ based diet, since its original collection from a commercial tomato greenhouse in British Columbia, Canada in 2001 (labeled T2c in [19]). Resistance to *Bt* was maintained by exposing larvae to 40 KIU ml^−1^ diet *Bt* subsp. *kurstaki* (DiPel 2x DF, Valent Biosciences, Libertyville, IL, USA) every generation. The *Bt*-susceptible colony derives from the *Bt*-resistant line but was reared without any *Btk* exposure [19]. Approximately 200 moths were mated each generation to minimize inbreeding. Back-crosses between the resistant and susceptible colonies were performed to homogenize their genetic background, and then re-selected with *Bt* so that the major genetic difference between the two colonies was likely to be *Bt* resistance [49]. For the purpose of this study, we used larvae from the *Bt*-susceptible line (Bt-S), and larvae from the *Bt*-resistant line that were not selected with *Bt* for one generation (Bt-RU) prior to the experiments to reduce trans-generational effects from the *Bt* selections [33].

### Artificial diets

Insects were routinely reared on the wheat-germ based diet (‘colony diet’) which they were originally established on. For the experiments, larvae were reared on the colony diet from egg-hatch until exposure to the wheat-germ free, nutritionally-defined treatment diets. The colony diet consisted of 60% protein and digestible carbohydrate, and had an approximate protein to digestible carbohydrate ratio of 1 to 1.1 (Bio-Serv, Frenchtown, NJ, USA). ‘Treatment diets’ were prepared according to Shikano and Cory [40]. They contained no wheat germ and consisted of dietary macronutrient ratios manipulated by altering the ratios of protein (casein) and digestible carbohydrate (sucrose) that made up 60% of the dry weight. The ratios were as follows (% protein: % carbohydrate): 50∶10, 40∶20, 30∶30, 20∶40, 10∶50. They were selected to encompass the wide range of protein and carbohydrate contents found in *T. ni* host plants. For example, between-species variation in protein and digestible carbohydrate content in nine Brassicacae species (preferred host plants of *T. ni*) ranged from 12–37% dry weight in protein (% nitrogen multiplied by conversion factor of 6.25 [50]) and 11–60% dry weight in digestible carbohydrate [50]. Protein content can likely reach up to 50% as supplementation of soil with nitrogen is known to increase protein content in a Brassicacae specie (eg. [51]). Other components of the dry diet included Wesson's salt (5%), cholesterol (1.5%), ascorbic acid (1%), sorbic acid (0.5%), sodium alginate (2.5%), sucrose-free Vanderzant vitamin mix (3.5%), wheat-germ oil (1%) and cellulose (25%). The diets were provided to larvae in 1.35% agar solution in a 5∶1 agar solution∶dry diet ratio.

### Feeding experiment

We followed the protocol of [52] with some minor modifications. Freshly moulted final (fifth) instar larvae (100 *Bt*-RU and 100 *Bt*-S) were weighed and individually fed a single pre-weighed block of one of five treatment diets in 30 ml plastic cups at 25°C and 16∶8 (L∶D) photoperiod. Uneaten diet was replaced with fresh diet blocks daily and frass was removed every day until pupation. Each day, the uneaten diet was dried to constant mass in a desiccating oven at 50°C for 24 hrs, then weighed to the nearest 0.1 mg. Pre-weighed diet blocks without larvae were run at the same time to construct a regression equation that was used to back calculate the initial dry mass of the diet blocks. Daily consumption was estimated by calculating the difference between the dry initial and final mass of the diet blocks. Only five Bt-S and one Bt-RU larvae either rejected the diet or failed to pupate and were removed from analyses. All pupae were weighed three days after pupal initiation then dried in a desiccating oven at 50°C for 48 hrs until constant mass. Dry initial larval mass, used to calculate relative growth rate (RGR), was estimated by using a regression equation constructed from weighing 20 live final instar larvae, freezing them at −20°C for 30 min, then re-weighing after drying in a desiccating oven at 50°C for 24 hrs. RGR  =  [ln(dry pupal mass) – ln(dry initial larval mass)]/days to pupation [53].

Lipid and nitrogen content of pupae were measured to determine the differences in nutrient use associated with *Bt* resistance. To measure pupal lipid content, dried pupae were lipid-extracted in three changes of chloroform every 24 hrs, then re-dried and re-weighed. Only subsets of the lipid-free pupae were analyzed for nitrogen content due to logistical constraints. A randomly selected subsample of ten lipid-free dry pupae from each diet treatment per colony was individually ground for 10 sec into a homogenous powder using a Mini-BeadBeater (BioSpec Products, Bartlesville, OK, USA). An approximately 2 mg powdered sample of each pupa was weighed to the nearest 0.001 mg and loaded onto an elemental analyzer (Vario Micro Cube CHNS Analyzer, Elementar Americas Inc.). Nitrogen content of each sample determined by the elemental analyzer was used to back-calculate and estimate nitrogen content in each pupa.

### Bt-challenge experiment

Freshly moulted fourth instar larvae were fed one of five diet treatments individually in cells of 48-well tissue culture plates until moulting to the fifth instar. Freshly moulted fifth instars were transferred to individual cells of 12-well tissue culture plates containing colony diet treated with one of five concentrations of *Bt* (DiPel) (0, 0.25, 0.5, 1, 2, 4 KIU ml^−1^ diet for Bt-S; 0, 10, 20, 40, 80, 160 KIU ml^−1^ diet for Bt-RU). Larvae were challenged with *Bt* on colony diet to equalize the *Bt* exposure between diet treatments. There were 48 larvae per concentration of *Bt* per treatment diet per colony. Larval mortality was recorded after three days on *Bt*-treated diet.

### Statistical analyses

Measures of diet consumption, pupal weight, relative growth rate, and pupal nitrogen and lipid content were analyzed using analysis of covariance (ANCOVA). Although female pupae tend to weigh less than male pupae, sex did not interact with P∶C ratio and colony. Therefore, sex was used as a covariate along with initial larval mass. Diet consumed was log transformed and relative growth rate was squared to meet the underlying assumptions of ANCOVA. Tukey HSD comparisons were performed when significant differences among treatments were detected. Time to pupation was analyzed using Cox proportional hazards regression model. For all analyses, all factors and their interactions were fitted initially in the model and non-significant terms were removed sequentially to produce the final minimal model. All figures are presented with the least squares means adjusted for sex and initial larval mass, and back transformed least squares means where transformations were used. Mortality data for the *Bt* assay were analysed using generalized linear models (GLM), using a binomial error structure and a logit link function. Chi-square pairwise contrasts were used to determine significant differences in mortality between diet treatments. As median lethal doses or concentrations are frequently used for comparison in the insect pathology literature, we also estimated the LC_50_ (concentration of *Bt* (DiPel) that killed 50% of exposed larvae plus its 95% confidence intervals) from the final, minimal GLM using the inverse prediction option in JMP. JMP (version 10, SAS Institute Inc., Cary, NC) was used for all statistical analyses. The dataset has been made available as supporting information (Dataset S1).

## Results

### Performance

**Figure 1:**
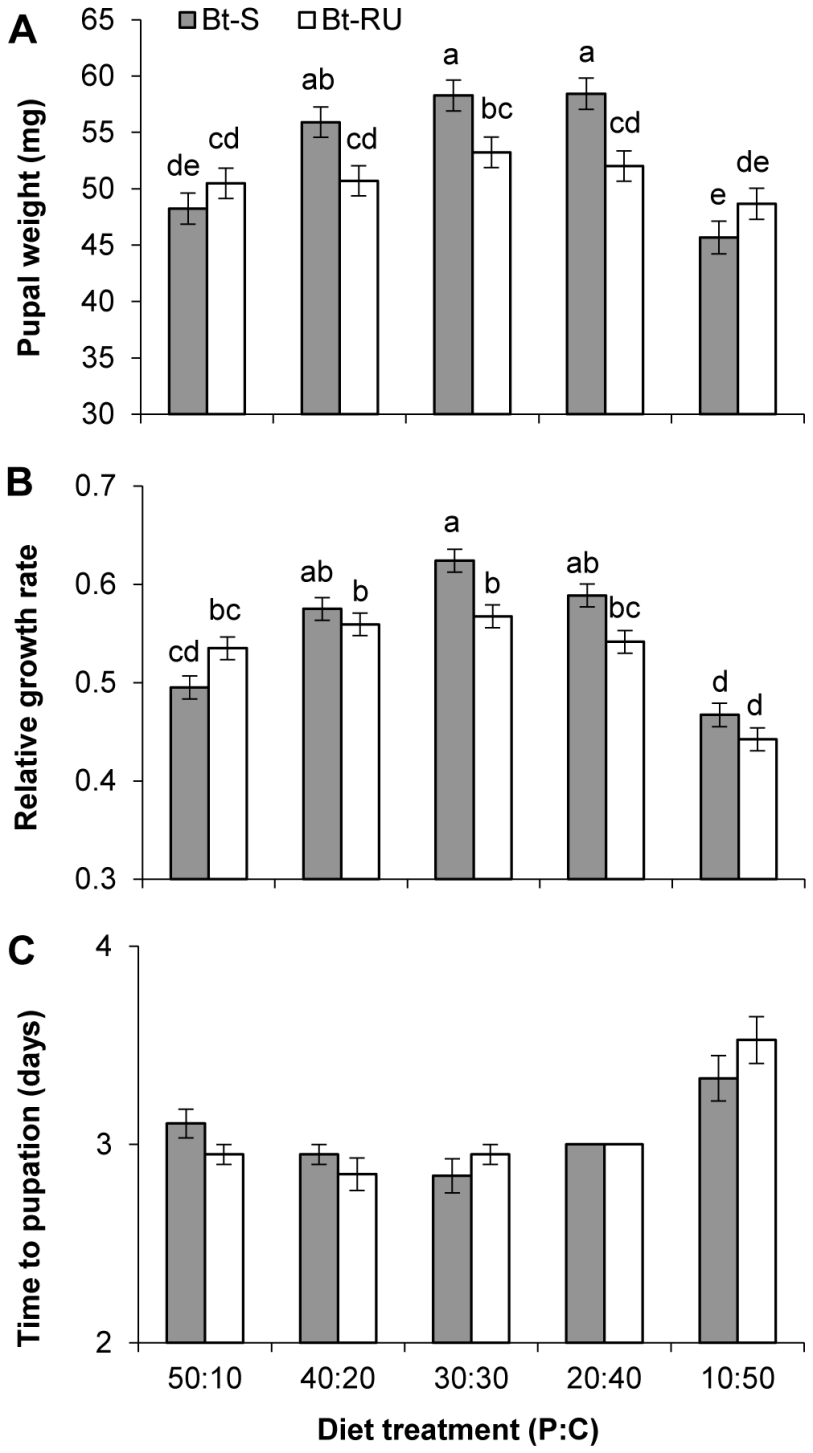
Fitness costs associated with *Bt* resistance in *T. ni*. (A) Pupal dry weight and (B) relative growth rate (RGR) for final instar Bt-S and Bt-RU larvae across the five P∶C ratio diets, presented as least squares means (±SE) adjusted for initial larval weight and sex. (C) Mean (±SE) number of days to pupation. RGR  =  [ln(dry pupal mass) – ln(dry initial larval mass)]/days to pupation [53]. Different letters indicate significant differences (Tukey HSD comparison).

Bt-RU individuals had significantly lower pupal mass than Bt-S insects; however, this varied with the dietary P∶C ratio, with the Bt-RU line being less affected by nutrient ratio (Figure 1A; Table 1). Bt-S pupae weighed more than Bt-RU on the three most balanced dietary P∶C ratios, but the mass of Bt-S pupae decreased on the two extreme P∶C ratio diets, resulting in similar masses for both lines. The relative growth rate (RGR) of Bt-S was also significantly faster than Bt-RU but this also varied with diet and only reached significance on the balanced diet (30p∶30c) (Figure 1B; Table 1). For Bt-RU, RGR was significantly lower on the most carbohydrate-rich diet relative to the other diets. For Bt-S, RGR was significantly lower on both the extreme diets, compared to the three most balanced diets. The two *T. ni* strains took the same time to reach the pupal stage (Figure 1C; Table 1) and time to pupation was only delayed on the most carbohydrate-rich diet for both strains.

**Table 1:** Analyses for pupal weight, larval growth rate, and days to pupation for *Bt*-resistant and susceptible *T. ni*.

	aPupal weight (n = 194)			aRelative growth rate (n = 194)			*Days to pupation (n = 194)		
	DF	*F*	*p*	DF	*F*	*p*	DF	*X* ^2^	*p*
Initial mass	1,182	26.42	**<0.001**	1,182	84.17	**<0.001**	1	3.64	0.06
Sex	1,182	13.81	**<0.001**	1,182	0.011	0.92	1	1.17	0.28
Colony	1,182	7.02	**<0.01**	1,182	7.88	**<0.01**	1	0.025	0.87
P∶C ratio	4,182	14.36	**<0.001**	4,182	35.21	**<0.001**	4	10.31	**0.04**
Colony × P∶C ratio	4,182	5.45	**<0.001**	4,182	4.86	**<0.01**	4	0.77	0.94

a Analyses of Covariance

* Cox proportional hazards regression model

Colony  =  Bt-S vs Bt-RU

Values in boldface are significant at *p*<0.05.

### Nutrient intake and efficiency of conversion into body mass

**Figure 2:**
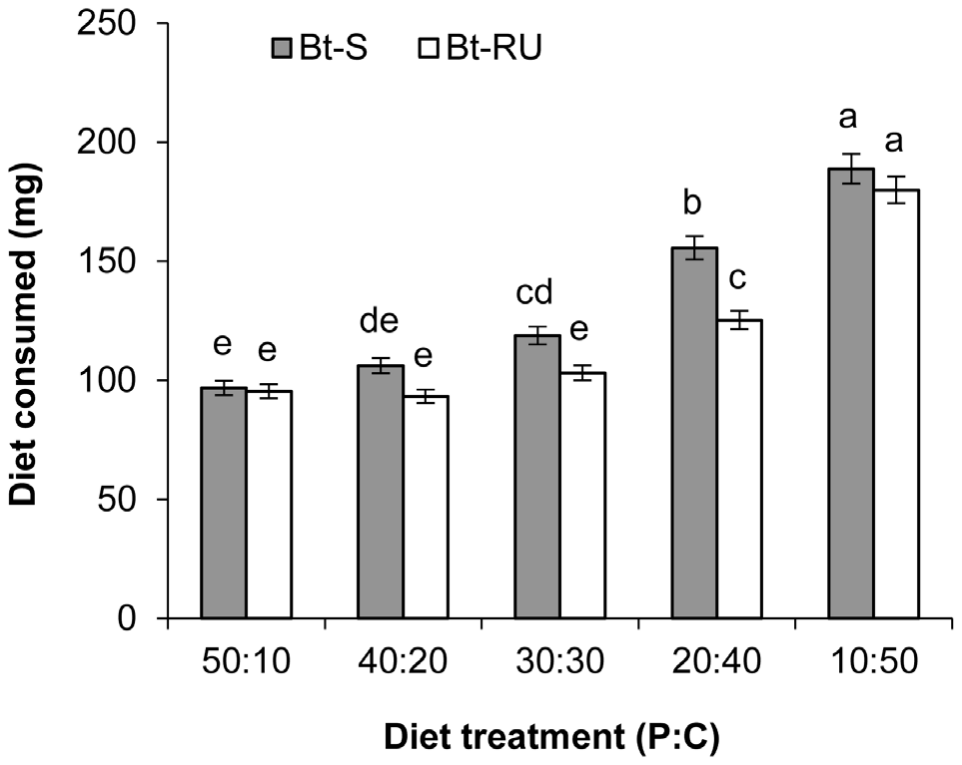
Fitness costs in *T. ni* are associated with reduced consumption. Diet consumption (protein and carbohydrate combined) by final instar Bt-S and Bt-RU larvae across the five P∶C ratio diets over the full larval stadium. Consumption is presented as least squares means (±SE) adjusted for initial larval weight and sex. Different letters indicate significant differences (Tukey HSD comparison).

Bt-RU consumed significantly less than Bt-S (Colony, *F*_1,182_ = 33.84 *p*<0.0001; Figure 2) and consumption increased as the P∶C ratio of the diet became increasingly carbohydrate-rich (P∶C ratio of diet, *F*_4,182_ = 160.21, *p*<0.001). The two insect lines responded differently to changing P∶C ratio and the reduced consumption of the resistant insects was primarily due to eating significantly less of the 30p∶30c and 20p∶40c diets (P∶C ratio by Colony, *F*_4,182_ = 3.54, *p* = 0.008). Initial mass of larvae had no effect on diet consumption (Initial mass, *F*_1,182_ = 0.14, *p* = 0.71), while males consumed more diet than females (Sex, *F*_1,182_ = 14.85, *p* = 0.0002).

**Figure 3:**
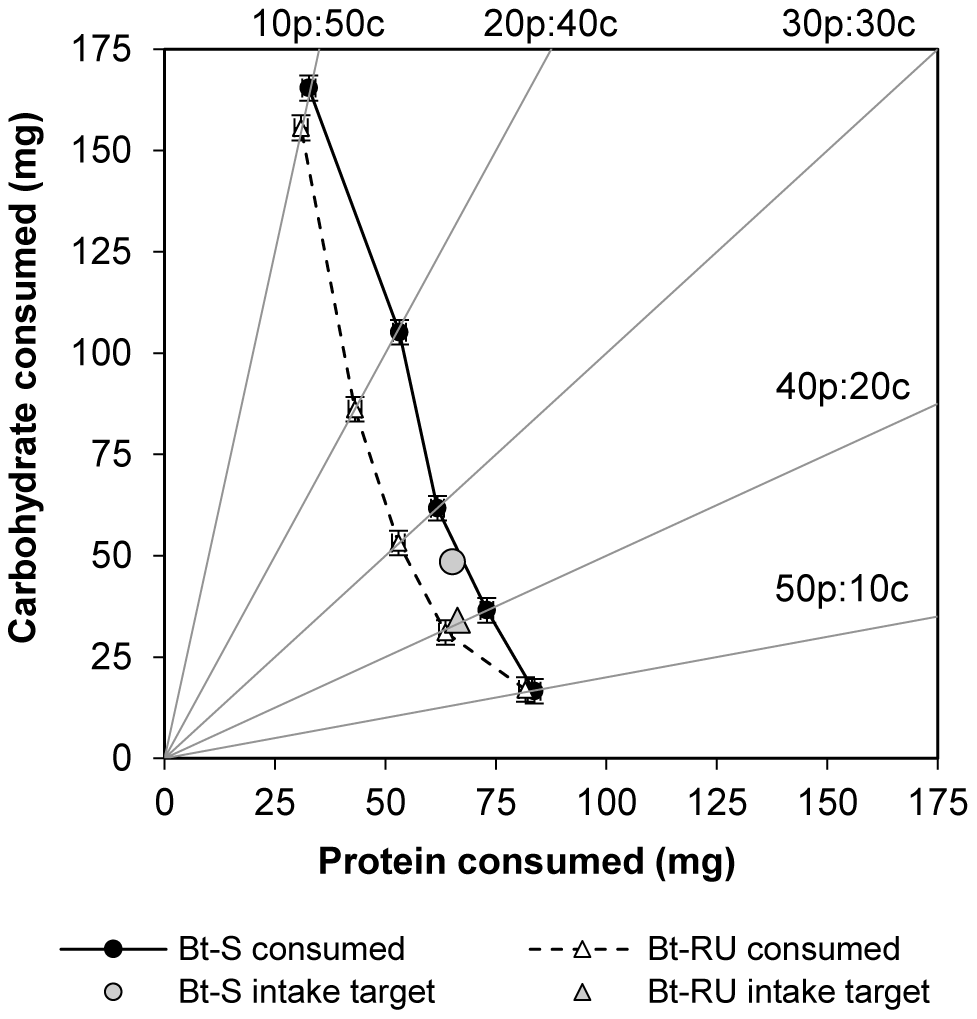
Compensatory protein-carbohydrate consumption. Bivariate least squares means (±SE) for protein and carbohydrate intake for final instar Bt-S and Bt-RU larvae adjusted for initial larval weight and sex. Points along each trajectory correspond to the cumulative intake of protein and carbohydrate over the entire final larval stadium. Solid gray lines represent nutrient ratios for the five food treatments (P∶C = 50∶10, 40∶20, 30∶30, 20∶40, 10∶50). Intake points for each day are connected by solid black lines and dashed black lines for Bt-S and Bt-RU respectively. Optimal intake targets (Bt-S, grey circle; Bt-RU, grey triangle) were obtained from Shikano and Cory [40].

Insects will select specific amounts of required nutrients to achieve an optimal blend of nutrients when they are given a choice of foods differing in nutritional content. This has been termed the intake target (see review by [48]). Here, *T. ni* larvae were prevented from reaching their intake target (obtained from [40]) because they were restricted to one of five P∶C ratios. When we examined the amount of protein and carbohydrate that they consumed on each P∶C ratio and compared it to the optimal amount that they required (intake target), we found that for both *T. ni* strains, carbohydrate consumption deviated more from the intake target than protein consumption (Figure 3). This suggests that maintaining protein intake close to their intake target is more important than maintaining carbohydrate intake.

**Figure 4:**
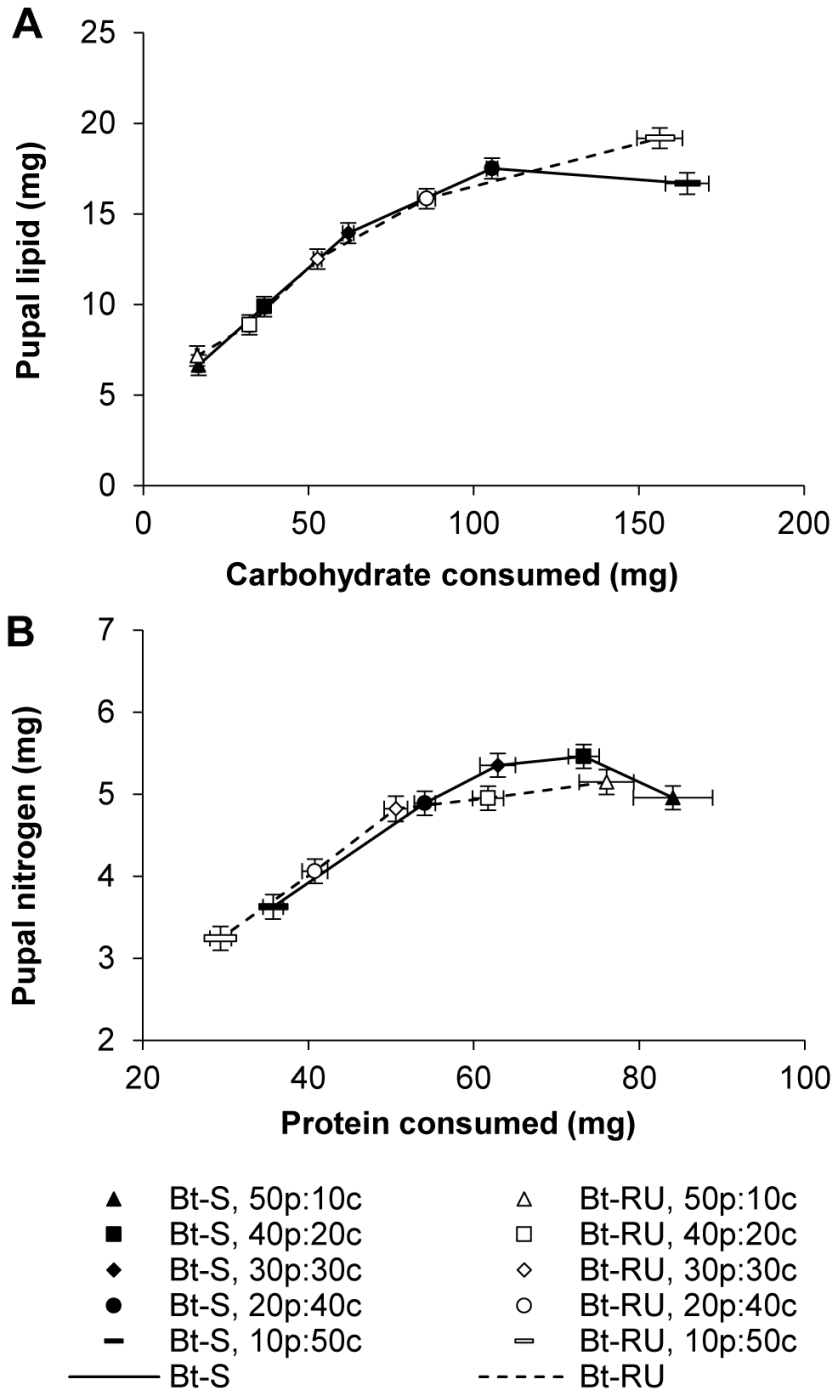
Bodily lipid and nitrogen accumulated across diet treatments. (A) Pupal lipid content plotted against carbohydrate intake and (B) pupal nitrogen content plotted against protein intake across the five P∶C ratio diets for final instar Bt-S and Bt-RU larvae. Data are presented as least squares means (±SE) adjusted for initial larval weight and sex.

Lipid and nitrogen content of the resulting pupae were plotted against the quantity of carbohydrate and protein ingested to assess the effect of macronutrient intake on body composition (Figure 4). Pupal lipid content increased and gradually came to a plateau as the P∶C ratio of the diet moved from protein-rich to carbohydrate-rich (P∶C ratio, *F*_4,182_ = 141.34, *p*<0.0001). Although overall lipid content did not differ between Bt-RU and Bt-S pupae (Colony, *F*_1,182_ = 0.38 *p* = 0.54), susceptible insects accumulated more lipid than resistant ones as the amount of carbohydrate in the diet increased; however, this trend was reversed on the most carbohydrate-rich diet (P∶C ratio by Colony, *F*_4,182_ = 4.75, *p* = 0.001). Both initial mass and sex affected total lipid content (Covariates: initial mass, *F*_1,182_ = 22.20, *p*<0.0001; sex, *F*_1,182_ = 14.40, *p* = 0.0002).

**Figure 5:**
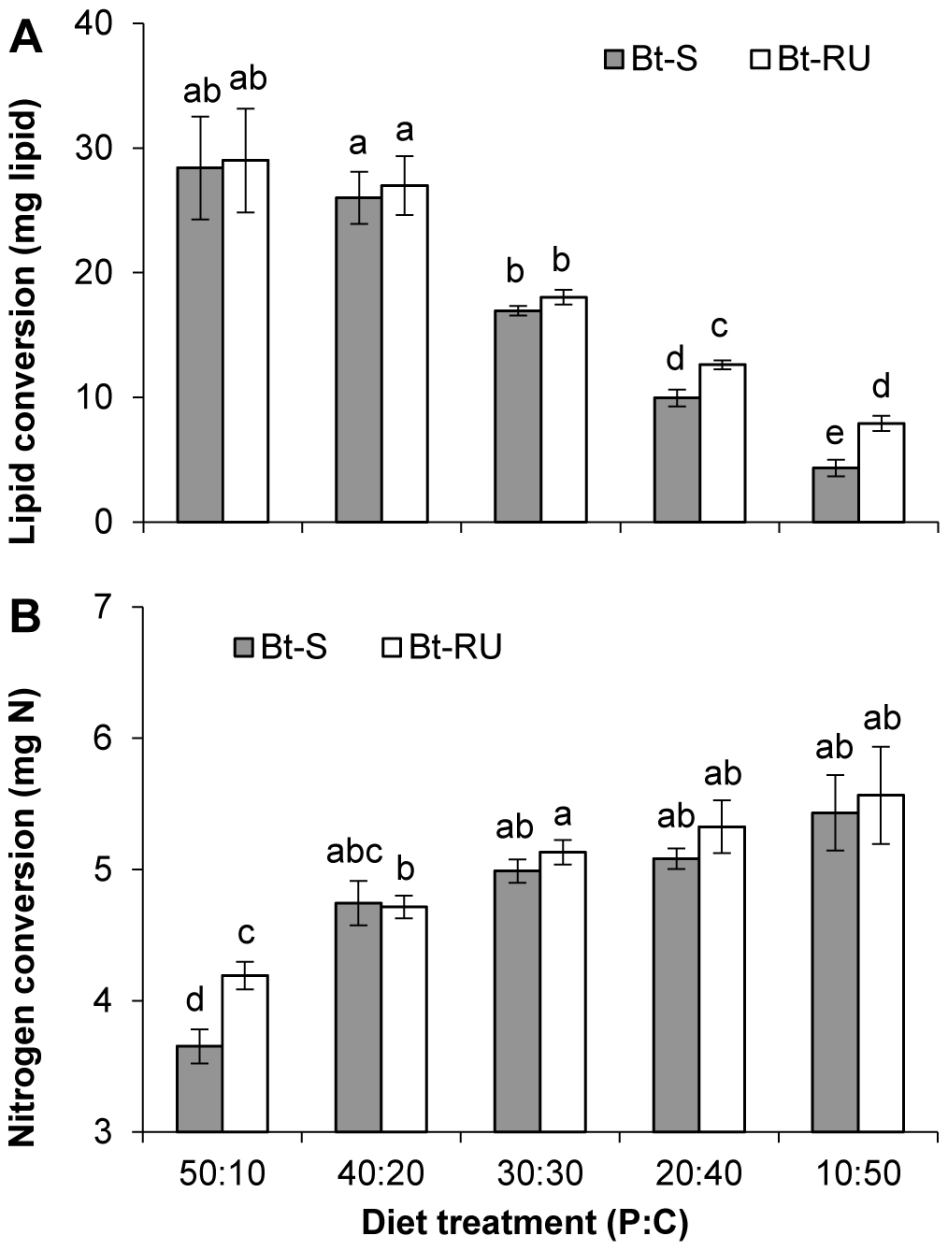
Comparisons of nutrient conversion efficiency between *Bt*-resistant and susceptible *T. ni*. (A) Efficiency of conversion of ingested carbohydrate to pupal lipid content and (B) ingested protein to pupal nitrogen content of pupated Bt-S (solid bars) and Bt-RU (open bars) across the five P∶C diet treatments. Efficiency of conversion of ingested macronutrients to body content are presented as least squares means of body content adjusted for the amount of ingested macronutrient (carbohydrate or protein), initial larval weight, and sex. Different letters indicate significant differences (Tukey HSD comparison).

To compare the efficiency of conversion of carbohydrate to lipid between colonies, we added the amount of carbohydrate consumed by each individual larva as a covariate. This adjusts the carbohydrate consumed, such that we can compare the amount of lipid that was produced if all larvae consumed the same amount of carbohydrate. The efficiency of conversion of carbohydrate to lipid decreased as the P∶C ratio of the diet became carbohydrate-rich (Figure 5A, Table 2). Furthermore, Bt-RU converted carbohydrate more efficiently into lipid than Bt-S on the two carbohydrate-rich diets.

**Table 2:** ANCOVA results comparing the efficiency of conversion of ingested carbohydrate into body lipid and ingested protein into body nitrogen between *Bt*-resistant and susceptible *T. ni* larvae.

	Lipid (n = 194)			Nitrogen (n = 100)		
Source	DF	F	p	DF	F	p
Initial larval weight	1,177	104.96	**<0.001**	1,83	33.71	**<0.001**
Sex	1,177	0.97	0.33	1,83	0.18	0.67
Colony	1,177	87.31	**<0.001**	1,83	10.12	**<0.01**
P∶C ratio	4,177	69.34	**<0.001**	4,83	27.16	**<0.0001**
Colony × P∶C ratio	4,177	10.85	**<0.001**	4,83	3.01	**0.02**
Covariate	1,177	213.54	**<0.001**	1,83	196.76	**<0.001**
Colony × Covariate	1,176	1.61	0.21	1,82	0.079	0.78
P∶C ratio × Covariate	4,177	21.55	**<0.001**	4,83	3.85	**<0.01**
Colony × P∶C ratio × Covariate	4,172	1.06	0.38	4,78	0.73	0.58

Colony  =  Bt-S vs Bt-RU

Covariate  =  Amount of carbohydrate or protein ingested.

Values in boldface are significant at *p*<0.05.

Pupal nitrogen content was consistent across P∶C ratios except on the most protein-poor diet (P∶C ratio of diet, *F*_4,88_ = 51.66, *p*<0.0001; Figure 4B). Overall, nitrogen content was significantly lower in Bt-RU than Bt-S pupae (Colony, *F*_1,88_ = 19.00 *p*<0.0001). The interaction between P∶C ratio and *Bt* resistance (P∶C ratio by Colony, *F*_4,88_ = 3.33, *p* = 0.01) indicates greater differences in nitrogen content between Bt-RU and Bt-S on the three more balanced diets with a significant difference on the 20p∶40c diet. The efficiency of conversion of protein to nitrogen decreased as the P∶C ratio of the diet became protein-rich (Figure 5B, Table 2). Furthermore, Bt-RU converted protein more efficiently than Bt-S on the most protein-rich diet.

### Bt-challenge

The composition of the diet consumed prior to *Bt* challenge altered the resulting mortality levels. Survival of Bt-S larvae increased significantly with increasing dietary P∶C ratio (Table 3; Figure S1; *X*_4_^2^ = 165.77, p<0.001). Mortality rose more rapidly with *Bt* concentration as the proportion of carbohydrate in the diet increased, but declined on the most carbohydrate-rich diet (10p∶50c) (*Bt* concentration by P∶C ratio, *X*_4_^2^ = 10.80, p = 0.03; Table 3; Figure S1). As expected, mortality increased with increasing *Bt* concentration (*X*_1_^2^ = 522.15, p<0.001). For Bt-RU, survival also increased with increasing P∶C ratio (*X*_4_^2^ = 62.34, p<0.001); however, in contrast to Bt-S, it declined sharply on the most protein-biased diet (by 44% based on LC_50_; Table 3) from its peak on the 40p∶20c diet (pairwise contrast between 40p∶20c and 50p∶10c, *X*_1_^2^ = 12.11, p<0.001). Again, mortality increased with increasing *Bt* concentration (*X*_1_^2^ = 337.15, p<0.001). There was no interaction between *Bt* concentration and diet (*X*_4_^2^ = 2.04, p = 0.73), indicating that while levels of mortality differed across P∶C ratio diets, the rate at which mortality increased with *Bt* concentration was the same on each diet (i.e. equal slopes). There was no control mortality for both Bt-S and Bt-RU.

**Table 3:** Median lethal concentration of *Bt* (Dipel; KIU ml^−1^ diet) for final instar Bt-S and Bt-RU larvae that were pre-fed one of five diets differing in P∶C ratios.

	Bt-S			Bt-RU			
P∶C ratio	LC_50_	(95% CI)	Slope	LC_50_	(95% CI)	Slope	*Resistance ratio
50p∶10c	2.05	(1.64–2.72)	3.24±0.50	21.80	(17.14–27.47)	3.06±0.20	10.61
40p∶20c	0.91	(0.76–1.09)	4.33±0.48	38.62	(30.78–48.44)	3.06±0.20	42.39
30p∶30c	0.67	(0.56–0.78)	5.24±0.56	31.83	(25.30–39.91)	3.06±0.20	47.77
20p∶40c	0.58	(0.49–0.68)	5.53±0.60	24.78	(19.57–31.15)	3.06±0.20	42.48
10p∶50c	0.33	(0.25–0.40)	4.16±0.56	10.93	(8.22–14.23)	3.06±0.20	33.34

*Resistance ratio  =  (LC_50_ of Bt-RU)/(LC_50_ of Bt-S) [15]

## Discussion

*Bt*-resistance in several species is associated with significant costs such as reduced pupal weight and slower growth rate. Our findings show that, in our strain of *T. ni*, these costs are incurred primarily by reduced food consumption and not impaired conversion of ingested nutrients into body mass. In fact, under certain dietary conditions, *Bt*-resistant larvae converted nutrients more efficiently than susceptible larvae. However, greater efficiency of converting protein into bodily nitrogen on the most protein-rich diet was associated with a significant increase in mortality compared to more balanced diets when challenged with *Bt*. This demonstrates a detrimental effect of consuming excess dietary protein in *Bt*-resistant insects.

Insects, like other animals, have evolved behavioural and physiological mechanisms to obtain an optimal mixture and blend of nutrients [48]. Here, we restricted *T. ni* larvae to one of five P∶C ratios so that they only had three options: (1) consume the diet until it meets its requirement for protein even though it takes in too much or too little of carbohydrate, (2) consume until it meets the requirement for carbohydrate while suffering an excess or deficit of protein, or (3) feed to an intermediate point where the excesses and deficits of both nutrients are less extreme [48]. As none of the P∶C ratio diets we provided exactly matched the intake target ratio of Bt-S and Bt-RU (although 40p∶20c was very close for Bt-RU), both strains of *T. ni* consumed excess amounts of the plentiful nutrient to obtain sufficient amounts of the deficient nutrient. This behaviour is typical of generalist herbivores [54]. Excess carbohydrate consumption was greater than excess protein consumption, resulting in higher variability in pupal lipid content, suggesting the greater importance of nitrogen regulation in its diet. This is consistent with nutrient regulation in other lepidopteran species [52], [54]–[56]. Generalists such as *T. ni* are likely to encounter dietary heterogeneity, and thus have flexible metabolic strategies to deal with nutrient imbalances [54]. In *T. ni*, nitrogen accumulation is likely to be regulated by a post-ingestive mechanism, as excess protein consumption did not result in continued increases in bodily nitrogen content [57], [58]. Contrary to our hypothesis, Bt-RU showed higher efficiency of conversion of dietary protein into bodily nitrogen on the extremely protein-rich diet. This was associated with no change in pupal mass on the protein-rich diet, whereas the pupal mass of susceptible *T. ni* was negatively affected. The higher conversion efficiency of protein to nitrogen by Bt-RU, coupled with the negative effects of excess protein on pupal mass of susceptible *T. ni*, could explain the higher protein to carbohydrate ratio selected by Bt-RU in our previous study [40].

Body lipid content increased consistently in both strains as the amount of carbohydrate ingested increased. However, the efficiency with which carbohydrate was converted to lipid decreased as dietary carbohydrate content increased. This indicates that *T. ni* larvae regulate body lipid content, possibly through a post-ingestive regulatory mechanism that releases overeaten carbohydrate from their body [52]. Interestingly, Bt-RU had a better conversion efficiency of carbohydrate to lipid on the carbohydrate-rich diets. Accumulating more body lipid can be advantageous as it can prolong survival during starvation [59]. *Bt* resistance in Bt-RU is not an all-or-nothing response. High concentrations of *Bt* still inhibit feeding and slow growth due to the damaging effects of *Bt* toxins on the insect gut. Therefore, surviving starvation could be an important component of *Bt* resistance in this strain.

However, this notion is contradicted by higher survival of both strains after *Bt*-challenge when pre-fed higher protein diets (except 50p∶10c for Bt-RU). The *Bt* formulation used in this study contains *Bt* spores in addition to toxins. Once the toxins breach the midgut, *Bt* spores act synergistically with the toxins by germinating in the insect haemolymph causing septicaemia [31], [32]. Studies on lepidopteran larvae have found altered immune activity following oral inoculations with formulations of *Bt* spores and toxins, such as increased phagocytic activity and encapsulation rate [60], and changes in haemocyte density and phenoloxidase activity [61]. Furthermore, inducing an immune response (rate of melanization reaction) with a low concentration of a *Bt* formulation was associated with a subsequent increase in survival to challenge with the same *Bt* formulation [29]. Since higher haemocyte densities [42], as well as higher antimicrobial, encapsulation and phenoloxidase activities [41] were found in the larval haemolymph of two *Spodoptera* species after consuming higher P∶C ratio diets, it is possible that *T. ni* that have fed on higher protein diets had higher baseline immune activity at the time of *Bt*-challenge.

We observed a 44% decrease in LC_50_ in Bt-RU on the highest protein diet, compared to a peak on the 40p∶20c diet, whereas the LC_50_ of Bt-S larvae continued to increase with protein levels, resulting in a dramatic decline in the resistance ratio (Table 3). Bt-S, however, had significantly lower pupal weight and growth rate on the most protein biased diet. Thus, excess dietary protein is deleterious to each *T. ni* line, but in different ways. This could be due to the higher costs of catabolizing excess ingested protein [55], [62]. Negative effects of excess dietary protein on performance have been observed in other lepidopteran species [63], [64], but this is the first study to show an increase in the susceptibility of an insect resistant to a microbial insecticide.

Lastly, the fitness costs associated with *Bt*-resistance in our *T. ni* strain were due to reduced food consumption rather than less efficient nutrient processing. Reduced consumption could result from a change in the feedback mechanism that involves peripheral contact chemoreception. It provides information about the nutrient content of food and chemical composition of the haemolymph, which reflects the quality and quantity of nutrient uptake and metabolic activities within the insect [58]. *Bt*-resistant *T. ni* have lower densities of haemocytes, and lower concentrations of protein, and phenoloxidase in the haemolymph than susceptible *T. ni* [61]. Lower requirements for these haemolymph components might influence the feedback mechanism, thereby reducing nutrient intake. However, it is important to keep in mind that nutrient intake is a dynamic process that will be influenced by environmental stressors. For example, our *Bt*-resistant *T. ni* were recently shown to increase food consumption and weight gain when fed artificial diet treated with low doses of *Bt* compared to a control [65].

An important limitation of the present study is the use of casein as the only protein source (from an animal) and sucrose as the only carbohydrate source as these are not representative of the variety of nutrients available to *T. ni* feeding on plants. Therefore, the critical question that remains concerns the extent to which macronutrient ratios affect the performance of *Bt*-resistant and susceptible *T. ni* on natural host plants, since most herbivorous caterpillars have access to a variety of plants and plant parts that vary in nutritional content [66]. More studies of fitness costs incorporating nutritional ecology using other *Bt* resistant strains are needed to determine the generality of our findings.

## Supporting Information

Figure S1 **Logit mortality of** ***Bt*** **-challenged** ***T. ni*** **pre-fed dietary treatments.** Variation in mortality of Bt-RU and Bt-S to *Bt* after pre-feeding on one of five P∶C ratio diets. Symbols show the actual data points (solid symbols, Bt-S; open symbols, Bt-RU) and lines (solid line, Bt-S; dashed line, Bt-RU) are the fitted models. The statistical analyses were performed separately for Bt-S and Bt-RU. Values of 0% or 100% are not represented in logits.
(PDF)

Dataset S1 **Data of nutrient intake and development measures.**
(XLS)
