# Supplementary material for: Dietary Mechanism behind the Costs Associated with Resistance to Bacillus thuringiensis in the Cabbage Looper, Trichoplusia ni
Source: PLoS One. 2014 Aug 29;9(8):e105864. doi: 10.1371/journal.pone.0105864 (PMC4149471; doi:10.1371/journal.pone.0105864)
Supplement: Figure S1 — Logit mortality of Bt -challenged T. ni pre-fed dietary treatments. Variation in mortality of Bt-RU and Bt-S to Bt after pre-feeding on one of five P∶C ratio diets. Symbols show the actual data points (solid symbols, Bt-S; open symbols, Bt-RU) and lines (solid line, Bt-S; dashed line, Bt-RU) are the fitted models. The statistical analyses were performed separately for Bt-S and Bt-RU. Values of 0% or 100% are not represented in logits. (PDF) [file pone.0105864.s001.pdf]

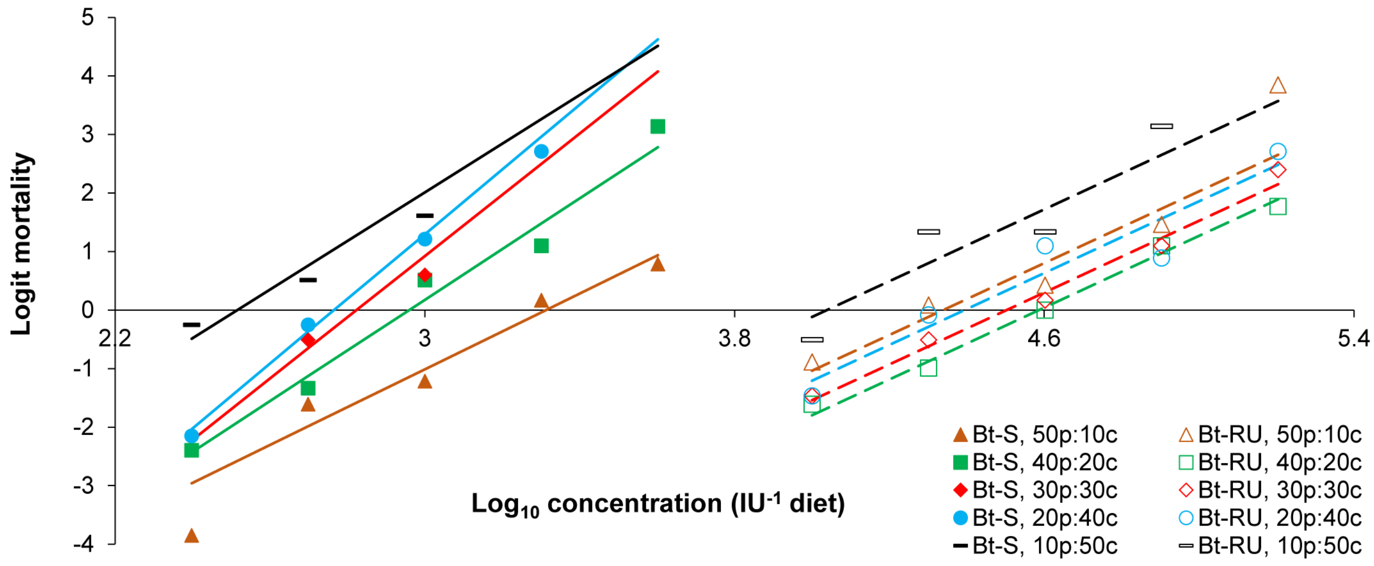

Figure S1. Diet composition before Bt-challenge affects survival. Variation in mortality of *Bt*-resistant and susceptible *T. ni* to *Bt* after pre-feeding on one of five P:C ratio diets. Symbols show the actual data points (solid symbols, Bt-S; open symbols, Bt-RU) and lines (solid line, Bt-S; dashed line, Bt-RU) are the fitted models. The statistical analyses were performed separately for Bt-S and Bt-RU. Values of 0% or 100% are not represented in logits.
